# Chinese medicine treatment of mastitis in COVID-19 patients: A protocol for systematic review

**DOI:** 10.1097/MD.0000000000021656

**Published:** 2020-08-28

**Authors:** Dengpeng Wen, Yu Shi, Xiaoxia Zhang, Gang Lv

**Affiliations:** a College of Acupuncture and Tuina, Chengdu University of Traditional Chinese Medicine, Chengdu, Sichuan; b Department of Breast and thyroid, Chongqing Traditional Chinese Medicine Hospital, Jiangbei District, Chongqing, China.

**Keywords:** Chinese medicine, COVID-19, mastitis, systematic review

## Abstract

**Background:**

Assessing the effectiveness and safety of Chinese medicine for the mastitis in COVID-19 patients is the main purpose of this systematic review protocol.

**Methods:**

The following electronic databases will be searched from inception to April 2020: MEDLINE, Ovid, EMBASE, the Cochrane Library, the Allied and Complementary Medicine Database (AMED), Chinese National Knowledge Infrastructure (CNKI), Chinese Biomedical Literature Database (CBM), VIP Database and Wanfang Database. In addition, Clinical trial registries, like the Chinese Clinical Trial Registry (ChiCTR), the Netherlands National Trial Register (NTR) and ClinicalTrials.gov, will be searched for ongoing trials with unpublished data. No language restrictions will be applied. The primary outcome will be the time of disappearance of main symptoms (including fever, asthenia, cough disappearance rate, and temperature recovery time), and serum cytokine levels. The secondary outcome will be the accompanying symptoms (such as myalgia, expectoration, stuffiness, runny nose, pharyngalgia, anhelation, chest distress, dyspnea, crackles, headache, nausea, vomiting, anorexia, diarrhea) disappear rate, negative COVID-19 results rate on 2 consecutive occasions (not on the same day), CT image improvement, average hospitalization time, occurrence rate of common type to severe form, clinical cure rate, and mortality. Two independent reviewers will conduct the study selection, data extraction and assessment. RevMan V.5.3 will be used for the assessment of risk of bias and data synthesis.

**Results:**

The results will provide a high-quality synthesis of current evidence for researchers in this subject area.

**Conclusion:**

The conclusion of the study will provide an evidence to judge whether Chinese medicine is effective and safe for mastitis in COVID-19 patients.

**PROSPERO registration number:**

CRD42020189924.

## 1 Introduction

Coronoviruses have been reported as causes of mild and moderate respiratory infections for over 50 years.^[1]^ Even though this group of viruses have been isolated from many different animals, bats are accepted major natural reservoir of coronaviruses.^[2,3]^ Four human coronavirus, 229E, HKU1, NL63, and OC43, are known as causes of common cold in humans.^[1]^ However, recently-detected coronaviruses, SARS CoV (2002), MERS-CoV (2012) completely altered all known approaches about this virus group because these viruses caused severe acute respiratory infections and nosocomial outbreaks. In the end of 2019, a novel coronavirus, now known as SARS-CoV-2 (2019), suddenly emerged in Wuhan, China. The World Health Organization declared that the epidemic is a public health emergency of international concern on January 31, 2020. As of April 16, 2020, the emerging coronavirus infection, COVID-19, has been spreading worldwide, causing over 2 million cases and over 137 thousand of death. The symptoms of COVID-19 infection appear after an incubation period of approximately 5.2 days.^[4]^ The period from the onset of COVID-19 symptoms to death ranged from 6 to 41 days with a median of 14 days.^[7]^ This period is dependent on the age of the patient and status of the patient's immune system. It was shorter among patients >70-years old compared with those under the age of 70.^[7]^ The most common symptoms at onset of COVID-19 illness are fever, cough, and fatigue, while other symptoms include sputum production, headache, hemoptysis, diarrhea, dyspnea, and lymphopenia.^[5–8]^ Mastitis is inflammation of breast tissue with or without microbial infection, breast mastitis often acute onset, mainly infectious inflammatory response. Non-lactation mastitis is a chronic onset of mass and pain. Traditional Chinese medicine is a treasure house for the Chinese nation to inherit for five thousand years and it has made a great contribution to the life and health of the Chinese nation. Traditional Chinese medicine stresses holistic view and dialectical theory of treatment. Traditional Chinese medicine (TCM) has a long history of treating mastitis and is effective.

Currently, there is a lack of evidence-based medical evidence for the treatment of covid-19 patients with mastitis. It is urgent for improvement of temper management, depression and quality of life in covid-19 patients with mastitis. It is necessary to make a systematic review to provide a convincing conclusion whether Chinese medicine is an appropriate method to treat COVID-19.

## 2 Methods and analysis

### 2.1 Study registration

This systematic review protocol has been registered with PROSPERO 2020 (registration number: CRD42020189924). And the protocol report is in the base of the preferred reporting items for systematic reviews and meta-analyses protocols (PRISMA-P) declaration guidelines.^[9]^ The review will be performed in line with the PRISMA declaration guidelines.^[10]^

## 3 Inclusion criteria for study selection

### 3.1 Type of study

All randomized controlled trials (RCTs) about Chinese medicine for mastitis in COVID-19 patients will be included. There is no language limitation. Non-RCTs, quasi-RCTs, case series, reviews, animal studies and any study with a sample size of less than ten participants will be excluded.

### 3.2 Type of participant

COVID-19 patients with mastitis, regardless of age, race or educational and economic status, will be included in the review.

### 3.3 Type of interventions

Experimental intervention will include Chinese medicine. Control interventions will be western medicine therapy.

### 3.4 Type of outcome measures

The primary outcome will be the time of disappearance of main symptoms (including fever, breast pain, cough disappearance rate, and temperature recovery time), and serum cytokine levels. The secondary outcome will be the accompanying symptoms (such as myalgia, expectoration, stuffiness, runny nose, pharyngalgia, anhelation, chest distress, dyspnea, headache, nausea, vomiting, anorexia, diarrhea) disappear rate, negative COVID-19 results rate on 2 consecutive occasions (not on the same day), CT image improvement, average hospitalization time, occurrence rate of common type to severe form, clinical cure rate, and mortality.

## 4 Search methods for identification of studies

### 4.1 Electronic data sources

The following electronic databases will be searched from inception to April 2020: MEDLINE, Ovid, EMBASE, the Cochrane Library, the Allied and Complementary Medicine Database (AMED), Chinese National Knowledge Infrastructure (CNKI), Chinese Biomedical Literature Database (CBM), VIP Database and Wanfang Database. In addition, Clinical trial registries, like the Chinese Clinical Trial Registry (ChiCTR), the Netherlands National Trial Register (NTR) and ClinicalTrials.gov, will be searched for ongoing trials with unpublished data. No language restrictions will be applied.

### 4.2 Searching other resources

A reference list of potential, qualified studies and related system reviews will be manually retrieved and reviewed. We will contact the author for the up-to-date data for the ongoing RCTs. Furthermore, relevant conference proceedings will be evaluated to identify studies related to this review.

### 4.3 Search strategy

The search strategy for PubMed is shown in Table 1. The following search keywords will be used: Chinese medicine (e.g., “traditional Chinese medicine” or “Chinese-medicine”; COVID-19 (e.g., “2019-nCoV” or “SARS-CoV-2” or “2019 novel coronavirus” or “COVID-19 virus” or “coronavirus disease 2019 virus” or “COVID19 virus”); Mastitis (e.g., “mammitis” or “mazoitis”) randomized controlled trial (e.g., “randomized controlled trial” or “controlled clinical trial” or “random allocation” or “randomized” or “randomly” or “double-blind method” or “single-blind method” or “clinical trial”.

**Table 1:**
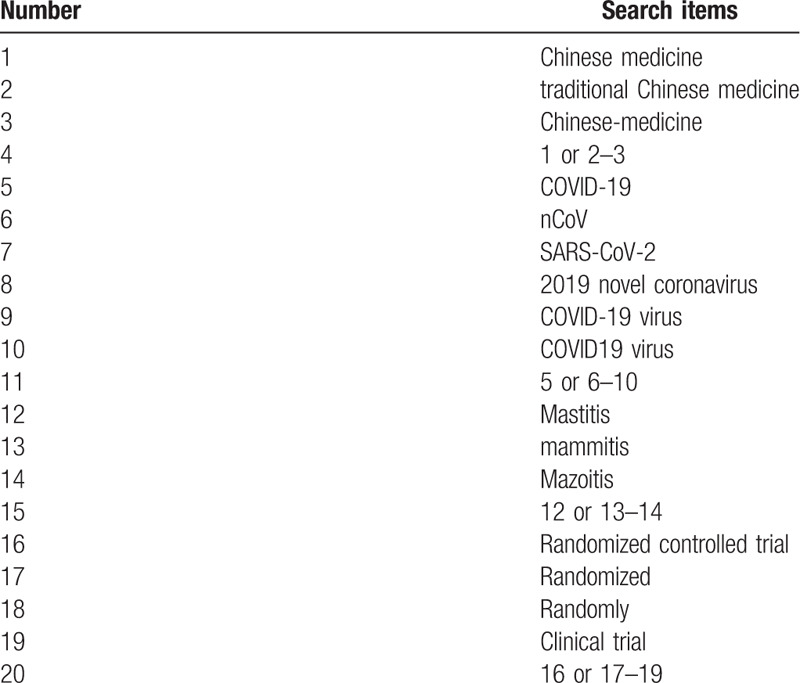
Search strategy for the PubMed database.

## 5 Data collection and analysis

### 5.1 Selection of studies

**Figure 1:**
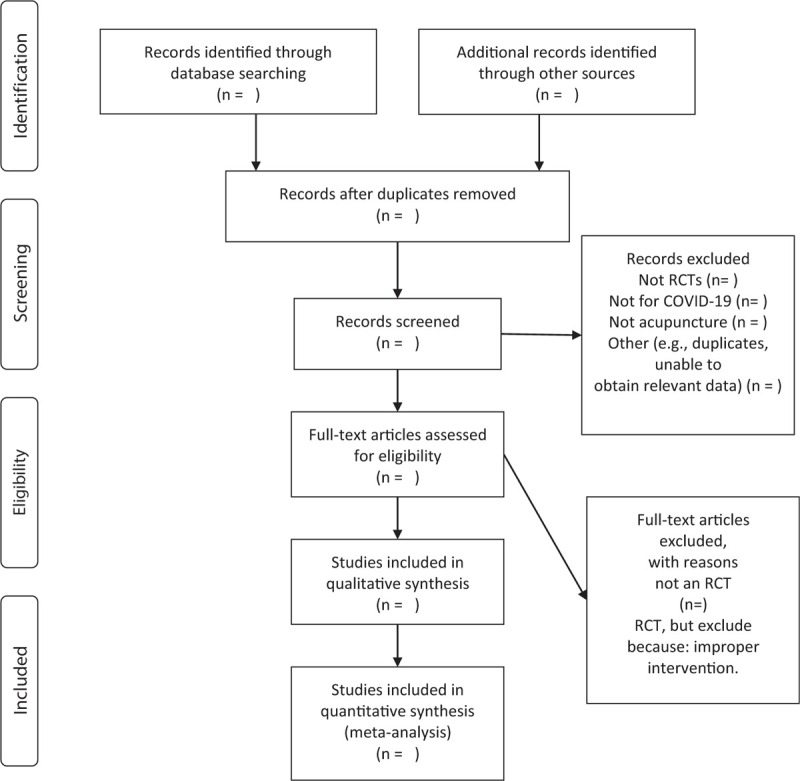
Flow chart of the study.

Two trained reviewers will review and screen the titles and abstracts of all searched studies independently. The duplicate records and ineligible studies will be eliminated to determine whether they meet the predefined inclusion criteria. A PRISMA flow diagram will be used to show the study selection process in Figure 1.

### 5.2 Data extraction and management

Two independent authors will extract data and fill in the data extraction form. These data will be obtained: general information, participants, methods, interventions, outcomes, results, adverse events, conflicts of interest, ethical approval and other information. If the data is insufficient, the authors will be contacted. Any disagreements will be resolved by discussion between the two authors, and further disagreements will be arbitrated by the third author.

### 5.3 Assessment of risk of bias and reporting of study quality

Two reviewers will independently access the quality of included literature and complete the Standards for Reporting Interventions in Clinical Trials of Acupuncture (STRICTA) checklist with the Cochrane collaboration risk-of-bias assessment method.^[11]^

### 5.4 Measures of treatment effect

RevMan V.5.3 will be used for data analysis and quantitative data synthesis. For continuous data, the mean difference (MD) or standard MD (SMD) will be used to measure the treatment effect with 95% CIs, if no heterogeneity is found. The random-effects model will be used if there is significant heterogeneity. A risk ratio (RR) with 95% CIs for analysis will be used for dichotomous data.

### 5.5 Unit of analysis issues

The units of each outcome from different trials will be converted to the International System of Units before statistical analysis.

### 5.6 Management of missing data

The authors will be contacted for the missing part. This will be documented and the available data will be extracted and analyzed if the missing data cannot be obtained.

### 5.7 Assessment of heterogeneity

I^2^ statistic will be used to quantify inconsistencies among the included studies. If the I^2^ value is less than 50%, it indicates that the studies have no significant statistical heterogeneity. And if the I^2^ value exceeds 50%, the studies are considered to have significant statistical heterogeneity among the trial and no meta-analysis need to be performed. We will conduct subgroup analysis to explore possible causes.

### 5.8 Assessment of reporting bias

Funnel plots will be used to access the reporting biases if there are over ten trials included in the meta-analysis.^[12]^

### 5.9 Data synthesis

RevMan V.53 will be used for data synthesis. The random-effects model will be used if the I^2^ value is no less than 50%. The fixed-effects model will be used if the heterogeneity tests show little statistical heterogeneity. If there is meaningful heterogeneity that cannot be explained by any assessment, meta-analysis will not be performed.

### 5.10 Subgroup analysis

There is no pre-subgroup plan. Subgroup analysis will be conducted if data are available. Factors such as different types of control interventions and different outcomes will be considered.

### 5.11 Sensitivity analysis

Sensitivity analysis will be conducted to test the robustness of the review conclusions if possible. We will evaluate the impacts of sample size, study design, methodological quality, and missing data.

### 5.12 Grading of evidence quality

The Grading of Recommendations Assessment approach will be used to judge the quality of the evidence for all outcomes.^[13]^ Risk of bias, heterogeneity, indirectness, imprecision and publication bias will be assessed. The assessments will be classified into 4 levels: high, moderate, low, or very low.

### 5.13 Ethics and dissemination

This protocol will not evaluate individual patient information or infringe patient rights and therefore does not require ethical approval. Results from this review will be disseminated through peer-reviewed journals and conference reports.

## 6 Discussion

This systematic review will be the first to assess the effectiveness and safety of Chinese medicine for mastitis in COVID-19 patients. The review contains four sections: identification, study inclusion, data extraction, and data synthesis. This review will aid doctors in the decision-making process for treating COVID-19 patients with mastitis, and will provide information for patients and health policy makers.

## Author Contributions

DPW and YS mainly contributed to this manuscript and joint first authors. GL obtained funding. XXZ and DPW drafted the protocol. YS make the search strategy and it will be conducted by them. XXZ and YS will obtain copies of the studies and screen the studies to be included. Data extraction from the studies will be done by YS. DPW will put the data into RevMan. Analyses will be conducted by YS.DPW and YS will draft the final review and XXZ will update the review. GL will act as an arbiter in the study selection stage. All authors have read and approved the final manuscript.
